# Altered gut microbiome in a mouse model of Gulf War Illness causes neuroinflammation and intestinal injury via leaky gut and TLR4 activation

**DOI:** 10.1371/journal.pone.0172914

**Published:** 2017-03-22

**Authors:** Firas Alhasson, Suvarthi Das, Ratanesh Seth, Diptadip Dattaroy, Varun Chandrashekaran, Caitlin N. Ryan, Luisa S. Chan, Traci Testerman, James Burch, Lorne J. Hofseth, Ronnie Horner, Mitzi Nagarkatti, Prakash Nagarkatti, Stephen M. Lasley, Saurabh Chatterjee

**Affiliations:** 1 Environmental Health and Disease Laboratory, Department of Environmental Health Sciences, Arnold School of Public Health, University of South Carolina, Columbia, South Carolina, United States of America; 2 Second Genome Inc., San Francisco, California, United States of America; 3 Department of Pathology, Microbiology and Immunology, University of South Carolina School of Medicine, Columbia, South Carolina, United States of America; 4 Department of Epidemiology and Biostatistics, Arnold School of Public Health, University of South Carolina, Columbia, South Carolina, United States of America; 5 Department of Drug Discovery and Biomedical Sciences, South Carolina College of Pharmacy, University of South Carolina, Columbia, South Carolina, United States of America; 6 Department of Health Services, Policy and Management, Arnold School of Public Health, University of South Carolina. Columbia, South Carolina, United States of America; 7 Department of Cancer Biology and Pharmacology, University of Illinois College of Medicine, Peoria, Illinois, United States of America; National Institutes of Health, UNITED STATES

## Abstract

Many of the symptoms of Gulf War Illness (GWI) that include neurological abnormalities, neuroinflammation, chronic fatigue and gastrointestinal disturbances have been traced to Gulf War chemical exposure. Though the association and subsequent evidences are strong, the mechanisms that connect exposure to intestinal and neurological abnormalities remain unclear. Using an established rodent model of Gulf War Illness, we show that chemical exposure caused significant dysbiosis in the gut that included increased abundance of phylum Firmicutes and Tenericutes, and decreased abundance of Bacteroidetes. Several gram negative bacterial genera were enriched in the GWI-model that included *Allobaculum sp*. Altered microbiome caused significant decrease in tight junction protein Occludin with a concomitant increase in Claudin-2, a signature of a leaky gut. Resultant leaching of gut caused portal endotoxemia that led to upregulation of toll like receptor 4 (TLR4) activation in the small intestine and the brain. TLR4 knock out mice and mice that had gut decontamination showed significant decrease in tyrosine nitration and inflammatory mediators IL1β and MCP-1 in both the small intestine and frontal cortex. These events signified that gut dysbiosis with simultaneous leaky gut and systemic endotoxemia-induced TLR4 activation contributes to GW chemical-induced neuroinflammation and gastrointestinal disturbances.

## Introduction

Prior research shows that, shortly after the first Persian gulf war (GW), a series of unique symptoms plagued many of the deployed veterans [1, 2]. These symptoms included chronic fatigue, gastrointestinal (GI) disturbances, and irritable bowel syndrome (IBS), in addition to post traumatic stress disorder and neurological abnormalities[1]. Many of these symptoms are difficult to categorize as they have no known cause, no objective findings on clinical examination, no diagnostic biomarkers, no known tissue pathology, and no curative therapy[3].

Well-conducted clinical studies and nationwide federal surveys provide documented evidence that exposure to GW agents such as pesticides and insect repellents, along with the consumption of nerve agent pre-treatment sets containing Pyridostigmine Bromide (PB), are among the major causative factors implicated in the etiopathogenesis of Gulf War Illness (GWI) [1, 4–8]. Likewise, epidemiological studies provide critical support for a significant biological gradient between the concentration and frequent use of PB pills and the increased severity of the illness [9]. Rodent studies in a GWI model showed corticosterone primed neuroinflammation [10]. Interestingly, exposure to PB, Permethrin and corticosterone has been found to cause oxidative stress in liver and neuronal cells [11–15]. Recent studies show an association between mood and cognitive dysfunction and hippocampal pathology is epitomized by decreased neurogenesis, partial loss of principal neurons, and mild inflammation in a model of GWI[16]. Further, results with an increase in soluble cytokine receptors and decrease in IL6 indicate that specific inflammatory proteins may be associated with brain structure and function as indexed by hippocampal volume and PTSD symptoms[17]. GWI networks had more abundant connections but were less organized. A detailed analysis indicated that IL-1alpha and CD2+/CD26+ nodes strongly influenced this characteristic modulation of B and T cell network motifs. This potentially heightened lymphocyte and HPA axis responsiveness to IL-1 stimulation in the context of a mixed Th1:Th2 immune signature supports an autoimmune component in GWI etiology[18]. With evidence of the exposure of these chemicals in the war theater through oral, nasal, and dermal routes, it is reasonable to predict that the above-listed exposures, either in unison or individually, can cause severe organ-specific or systemic oxidative stress and inflammation, effects that may depend on the route of exposure.

Chemical exposures through an oral or systemic route have been shown to cause significant inflammation and intestinal oxidative stress[19–21]. GI disturbance in GWI closely resembles inflammatory bowel disease/syndrome[2]. Although there is no significant evidence of oxidative stress-mediated changes in the gut microbiome, intestinal inflammation associated with IBS, alcoholic steatohepatitis, obesity, nonalcoholic fatty liver disease and metabolic syndrome, they have been strongly linked to alterations in the gut microbiome[22–29]. There are at least 3000 species of bacteria residing in the human gut, and there is a unique prototype of bacterial diversity in every individual[30]. The last decade saw a tremendous increase in our understanding of how gut bacteria participate in health and disease. Significant areas in which gut bacteria have been found to play a role are obesity, metabolic syndrome and inflammatory bowel syndrome[30]. There is increasing evidence that the gut microbiome plays a role in modulating the neuronal responses[31]. Studies have shown that IBS patients are characterized by a reduction of butyrate-producing bacteria, known to improve intestinal barrier function, as well as a reduction of methane-producing microorganisms. This is a major mechanism of hydrogen disposal in the human colon, which could explain the excess of abdominal gas in IBS[32]. Significant data from studies of obese, metabolic syndrome and nonalcoholic fatty liver disease (NAFLD) patients have identified an increase in phylum Firmicutes over Bacteriodetes, which is otherwise a dominating phylum in normal, healthy individuals[22, 23]. On a family level, increases in Bacteriodaceae, Porphyomonadaceae, Prevotellaceae, Clostridiaceae and Enterobacteriaceae have been associated with adverse outcomes, while a decrease of Lactobacilaceae was seen in patients with IBS and NAFLD[24, 26, 33–35]. GWI is also characterized by systemic rise of pro-inflammatory cytokines, including TNF-α and IL1β[18, 36–38]. Interestingly, IBS, Obesity, NAFLD and nonalcoholic steatohepatitis have a significant pro-inflammatory component that includes macrophage activation, and triggering of toll-like receptor pathway. Several research reports also found strong evidence of a decrease in expression of gut junction proteins leading to portal endotoxemia[39–42]. With sufficient evidence from GW veteran studies about the existence of chronic fatigue and GI disturbances coupled with neuronal inflammation, it is all the more important that we explore newer and novel mechanistic pathways, especially the role of the gut microbiome in modulating adverse outcomes in GWI such as neuroinflammation and intestinal injury[2].

The present study examines the effect of exposure of gulf war chemicals PB and Permethrin and their resultant alteration of the gut microbiome as a key pathway to neuroinflammation and GI disturbances via systemic endotoxemia and toll like receptor pathway activation. This research that uses an established GWI rodent model, antibiotic treatment regimen (to nullify the effect of gut bacteria) and transgenic TLR4 knockout mice show a distinct role of the altered gut microbiome in causing inflammatory intestinal injury and neuronal inflammation.

## Materials and methods

### Materials

Neomycin trisulfate salt (Neomycin), Enrofloxacin, Pyridostigmine bromide (PB), Permithrin, lipopolysaccharides (LPS) and Corticosterone were purchased from Sigma- Aldrich (St. Louis, MO). Anti- claudin-2, anti- Occludin, anti-TLR4, anti-flotillin, anti 3-nitrotyrosine (3NT), anti-IL1β, and anti-MCP-1 primary antibodies were purchased from Abcam (Cambridge, MA). Species specific biotinylated conjugated secondary antibodies and Streptavidin-HRP (Vectastain Elite ABC kit) was purchased from Vector Laboratories (Burlingame, CA). Fluorescence conjugated (alexa fluor) secondary antibodies, ProLong Gold antifade mounting media with DAPI and Pierce LAL chromogenic endotoxin quantitation kit was bought from Thermo Fisher Scientific (Waltham, MA). All other chemicals which were used in this project purchased from sigma only if otherwise specified. Paraffin-embedding of tissue sections on slides were done by AML laboratories (Baltimore, MD). Microbiome analysis was done by Second Genome, the microbiome company (San Francisco, CA).

### Animals

Adult wild type male (C57BL/6J mice) and adult mice that contained the disrupted TLR4 gene (TLR4 KO)(B6.B10ScN-Tlr4^*lpsdel*^/JthJ) were purchased from the Jackson Laboratories (Bar Harbor, ME). Mice were implemented in accordance with NIH guideline for human care and use of laboratory animals and local IACUC standards. All procedures were approved by The University of South Carolina at Columbia, SC. Mice were housed individually and fed with chow diet at 22–24°C with a 12-h light/ 12-h dark cycle. All mice were sacrificed after animal experiments had been completed. Right after anesthesia, blood from the mice was drawn using cardiac puncture, in order to preserve serum for the experiments. The mice brain was removed immediately after terminal surgery. Olfactory bulbs (OB) and Frontal cortex (FC) were dissected out and were fixed by using Bouin's fixative solution. Fecal pellets and luminal contents were collected from the animals, followed by dissection of small intestine and colon. The tissues were fixed using 10% neutral buffered formalin. Distal segments of small intestines were used for the staining and visualizations.

### Chemical exposure and rodent model of Gulf War Illness

Mice were exposed to gulf war chemicals based on established rodent models of Gulf War Illness with some modifications[7, 10]. The treated mice group (GW-T) and TLR4 KO (GW-TLR4 KO) mice group were dosed tri-weekly for one week with PB (2mg/kg) and Permethrin.(200 mg/kg) via oral route. After completion of PB/Permethrin dosages, mice were administered corticosterone intraperitoneally with a dose of 100μg/mice/day for 5 days of the week for one week. The dose of corticosterone was selected from the study which exposed mice to 200mg/L of corticosterone through drinking water. The i.p dose of corticosterone had similar immunosuppression as examined by low splenic T cell proliferation (data not shown).). Similarly, another group of mice (GW-Ab) were exposed to PB/Permethrin and corticosterone as above mentioned dosages along with antibiotics (Neomycin 45 mg/kg and Enrofloxacin 1mg/kg) thrice per week for two weeks for intestinal decontamination. The control group (GW-Con) of mice was received saline injections and vehicle for oral gavage in the same paradigm.

### Lipopolysaccharides (LPS) exposure

In order to generate TLR4 activated positive control, wild type male (C57BL/6J) mice were exposed to LPS as published earlier[43]. Briefly, mice received single bolus infusion of LPS (12 mg/kg) and mice were euthanized after 24 h for tissue and serum. A control group was also included, where normal mice received saline in place of LPS. LPS was dissolved in pyrogen-free saline and administered through the intraperitoneal (i.p.) route.

### Microbiome analysis

Fecal pellets and luminal contents were collected from the animals of each group after sacrifice, and then sent to Second Genome, for microbiome analysis. Second Genome performed nucleic acid isolation with the MoBio PowerMag® Microbiome kit (Carlsbad, CA) according to manufacturer’s guidelines and optimized for high-throughput processing. For detailed methods used at Second Genome, refer to supplementary methods.

### Laboratory methods

#### Immunohistochemistry

The brain and small intestine were collected from mice and fixed in Bouin's solution and 10% neutral buffered formalin respectively. The fixed tissues were embedded in paraffin and cut in 5 μM thick section. These sections were subjected to deparaffinization using standard protocol [44]. Epitope retrieval solution and steamer (IHC-Word, Woodstock, MD) were used for epitope retrieval for deparaffinized sections. 3% H_2_O_2_ was used for the recommended time to block the endogenous peroxidase. After serum blocking, the primary antibodies such as IL1β and MCP-1 were used in recommended concentrations. Species- specific biotinylated conjugated secondary antibodies and streptavidin conjugated with HRP were used to implement antigen specific immunohistochemistry. 3,3'-Diaminobenzidine (DAB) (Sigma Aldrich, St Louis, MD) was used as a chromogenic substrate. Mayer's Hematoxylin solution (Sigma Aldrich) was used as a counter stain. Sections were washed between steps using phosphate buffered saline 1X. Finally, stained sections were mounted in Simpo-mount (GBI laboratories, Mukilteo, WA). Tissue sections were observed using Olympus BX51 microscope (Olympus, America). Cellsens software from Olympus America (Center Valley, PA) was used for morphometric analysis of images.

#### Immunofluorescence staining for microscopy

Paraffin embedded sections were deparaffinized using standard protocol. Epitope retrieval solution and steamer were used for epitope retrieval of sections. Primary antibodies such as Claudin-2, Occludin, 3NT, TLR4, and Flotillin were used at recommended dilutions. Species specific secondary antibodies conjugated with Alexa Fluor (633-red and 488-green) were used at advised dilution. In the end, the stained sections were mounted using prolong gold antifade reagent with DAPI. Sections were observed under–Olympus florescence microscope using 20X, 40X objective lens. -

#### LAL assays

Serum from mice were diluted 1:50 and used for quantifying the portal endotoxin concentration, using the Pierce LAL chromogenic endotoxin quantitation kit (following manufacturer’s protocol)

#### Western blot

25 mg of tissue from each small intestinal sample was immediately homogenized in 250 μl of RIPA buffer with protease and phosphatase inhibitors cocktail (Pierce, Rockford, IL) using slow speed (to avoid frothing) mechanical homogenizer. The homogenate was centrifuged and the supernatant was collected and saved for experimental use. 30 μg of denatured protein from each sample was loaded per well of novex 4–12% bis-tris gradient gel (Life technologies, Carlsbad, CA) and subjected for standard SDS-PAGE. Separated protein bands were transferred to nitrocellulose membrane using precut nitrocellulose/filter paper sandwiches (Bio-Rad, Hercules, CA) and Trans–Blot Turbo transfer system (Bio-Rad) using 30 minutes transfer protocol. Further, blots were blocked with 5% non-fat milk solution prepared in Tris-buffered saline with 0.05% tween-20 (TBS-T). Primary antibodies were used at recommended dilutions in blocking buffer and incubated 2h at room temperature or overnight at 4°C. Species-specific (source of primary antibody) anti-IgG secondary antibody conjugated with HRP were used at recommended dilutions in blocking buffer and incubated 2h at room temperature. Pierce ECL Western Blotting substrate (Thermo Fisher Scientific Inc., Rockford, IL) was used in dark to develop the blot. Finally, the blot was imaged using G:Box Chemi XX6 (Syngene imaging systems) and subjected to densitometry analysis using Image J software.

### Statistical analysis

Prior to initiation of the study, we conducted calculations for each experimental condition with appropriate preliminary data to confirm that the sample number is sufficient to achieve a minimum statistical power of 0.80 at an alpha of 0.05. Student’s t-test was used to compare means between two groups at the termination of treatment. One way analysis of variance (ANOVA) was used with post-hoc comparisons among different exposure conditions or treatments (e.g., least significant differences or LSD statistic) to compare means among multiple groups at the end of the 2-week treatment period. A one-way ANOVA- was applied as needed, to evaluate differences among treatment groups. A Neuman-Keuls post-hoc test was carried out following ANOVA application. We used standard procedures for evaluating immunohistochemistry and relative mRNA expression data, as described in our previous publications.

## Results

### Gulf war chemical exposure and chronic corticosterone administration causes gut dysbiosis

**Fig 1:**
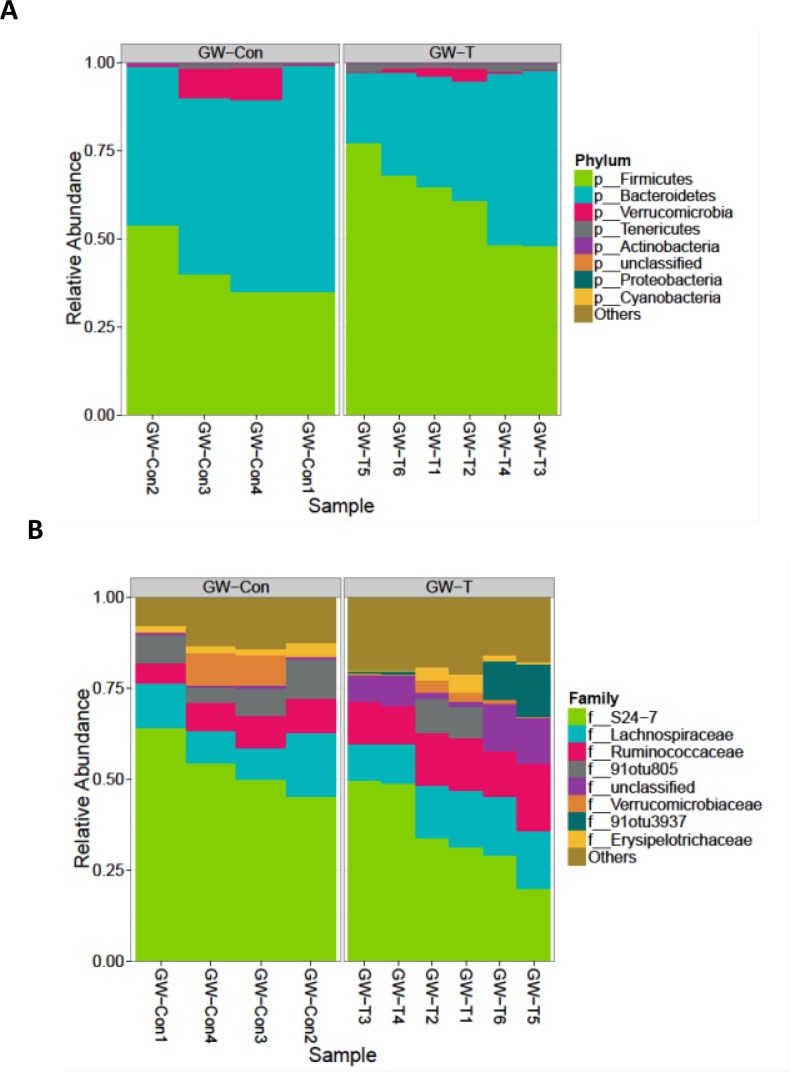
Gulf war chemical exposure alters gut microbiome. A. Proportional abundance of phyla: Graphical representation of the most abundant taxa of bacteria at the phylum level. Groups compared are gulf war chemical exposed group (GW-T) and control group fed with vehicle (GW-Con). -. Groups include individual samples numbered at the time of V4 16S rRNA sequencing. GW-T show marked higher percent relative Phylum-level abundance of Firmicutes, and Tenericutes over Bacteroidetes (KW p-value: 0.03) compared to GW-Con. B. Proportional abundance of families: Graphical representation of most abundant taxa at the family level in GW-T compared to GW-Con groups. GW-T show a significant increase in Ruminococcaceae and 2 other unclassified/unnamed Operational taxonomic units (OTUs) (KW p-value: 0.01) All profiles are inter-compared in a pair-wise fashion to determine a dissimilarity score and store it in a distance dissimilarity matrix. Distance functions produce low dissimilarity scores when comparing similar samples. Abundance-weighted sample pair-wise differences were calculated using the Bray-Curtis dissimilarity. Bray-Curtis dissimilarity is calculated by the ratio of the summed absolute differences in counts to the sum of abundances in the two samples (Bray and Curtis 1957). The binary dissimilarity values were calculated with the Jaccard index. This metric compares the number of mismatches (OTUs present in one but absent in the other) in two samples relative to the number of OTUs present in at least one of the samples (Jaccard 1912). Kruskal-Wallis rank sum test on top 8 most abundant phyla. Percent relative abundance means are provided.

**Fig 2:**
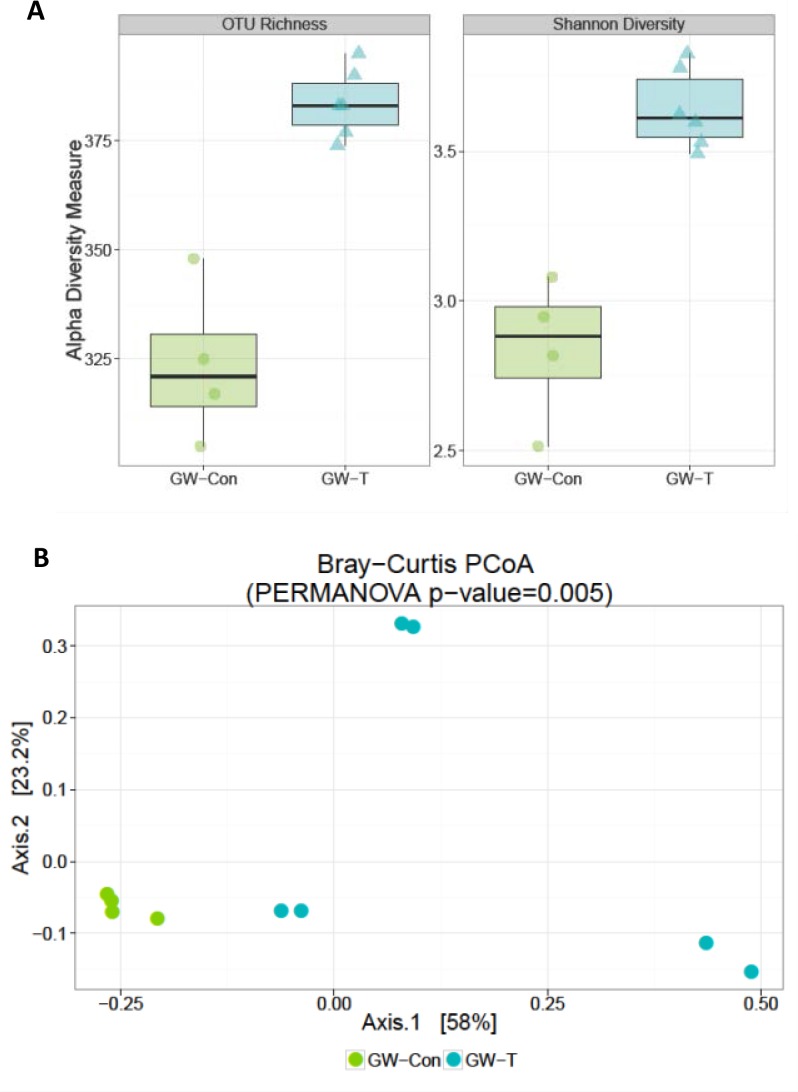
Alpha diversity estimates: Gulf war chemical exposure alters bacterial diversity in the intestinal lumen while there is no change among the individual groups themselves A. Left panel: OTU richness. Graphical representation of the number of OTUs present in each sample. Chao1 calculates the estimated sample richness (number of OTUs) based on sequencing depth and taking into account rare taxa that may be present in a sample GW-T has significant increase in OTU-richness compared to GW-Con (KW p-value: 0.01). Right panel: Graphical representation of Shannon diversity differences between GW-T and GW-Con. Shannon diversity utilizes the richness of a sample along with the relative abundance of the present OTUs to calculate a diversity index. There is an observed significant increase in Shannon diversity of GW-T over GW-Con (KW p-value: 0.01) B. Weighted ordination of GW-Con and GW-T groups. Dimensional reduction of the Bray-Curtis distance between microbiome samples, using the PCoA ordination method. Samples were separated according to groups along Axis 2 (PERMANOVA p-value = 0.005).

**Fig 3:**
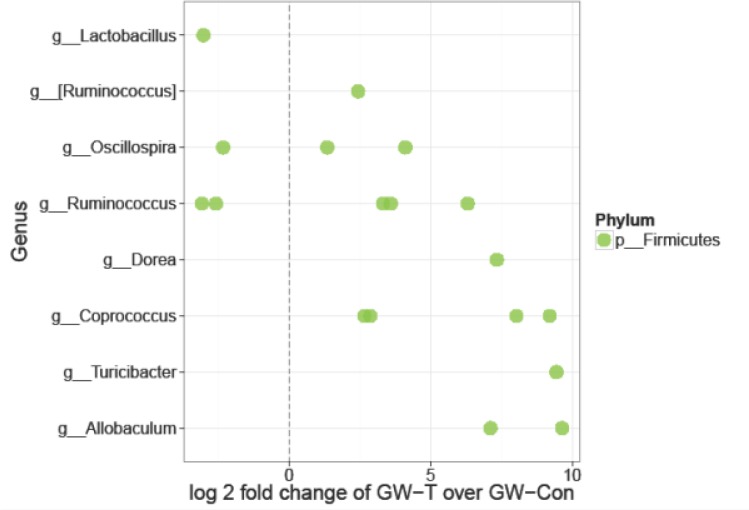
Differentially abundant features in GW-T vs. GW-Con: Each point represents an OTU belonging to each Genus. Features were considered significant if their FDR-corrected p-value was less than or equal to 0.05, and the absolute value of the Log-2 fold change was greater than or equal to 1. There were 109 significantly different features detected out of 577 tests. Only 18 features that were able to be classified at the genus level are shown in the plot.

Chemical exposures and chronic drug use cause significant oxidative stress in the liver, intestine and the kidneys signifying that the drugs and their intermediates might also have a cytotoxic effect on the normal gut microbial flora [44–47]. To study whether exposure of gulf war chemicals and the resultant chronic stress could alter the gut microbiome in the GWI-mice model, intestinal contents (illeal and cecal segments) and fecal pellets were analyzed for gut microbiome diversity by 16s V4 sequencing. Results showed that there was a significant differences in the composition of gut bacteria both at the phylum and family levels (Fig 1). GW chemical exposed group (GW-T) showed significantly higher percent relative Phylum-level abundance of Firmicutes -and Tenericutes over the Bacteroidetes (Kruskal-Wallis, KW signed rank p-value = 0.03, Table A in S1 File) as compared to the unexposed group (GW-Con, Fig 1A). GW-T also showed a significant increase in Ruminococcaceae and two other unclassified/unnamed families from the order Clostridiales (KW p-value = 0.01, Table B in S2 File). There was a reported decrease in abundance of family-level OTU S24-7. (KW p-value = 0.03,.Table B in S2 File) (Fig 1B), Gulf war chemical exposure altered bacterial alpha diversity in the intestinal lumen. GW-T had significant increase in OTU-richness compared to GW-Con (KW p-value: 0.01, Fig 2A left panel, Table C in S2 File) There was a significant increase in Shannon diversity of GW-T over GW-Con (KW p-value: 0.01, Fig 2A right panel, Table C in S2 File). There were significant differences in microbiome beta diversity between the two groups (PERMANOVA p-value of 0.005, Fig 2B). There were 109 significantly different Operational Taxonomic Units (OTUs) out of a total 577 OTUs between GW-T and GW-Con. only 18 OTUs that were able to be classified at the genus level were shown in the plot (Fig C, in S1 File). Certain OTUs representing the genera *Allobaculum*, *Coprococcus*, *Dorea*, *Turibacter and Ruminococcus* were abundant in GW-T, compared to GW-Con, the latter showing either fewer or no OTUs classifiable (attributable) to the same genera (Fig 3).

### Altered gut microbiome modulates tight junction protein levels of Claudin-2 and Occludin

**Fig 4:**
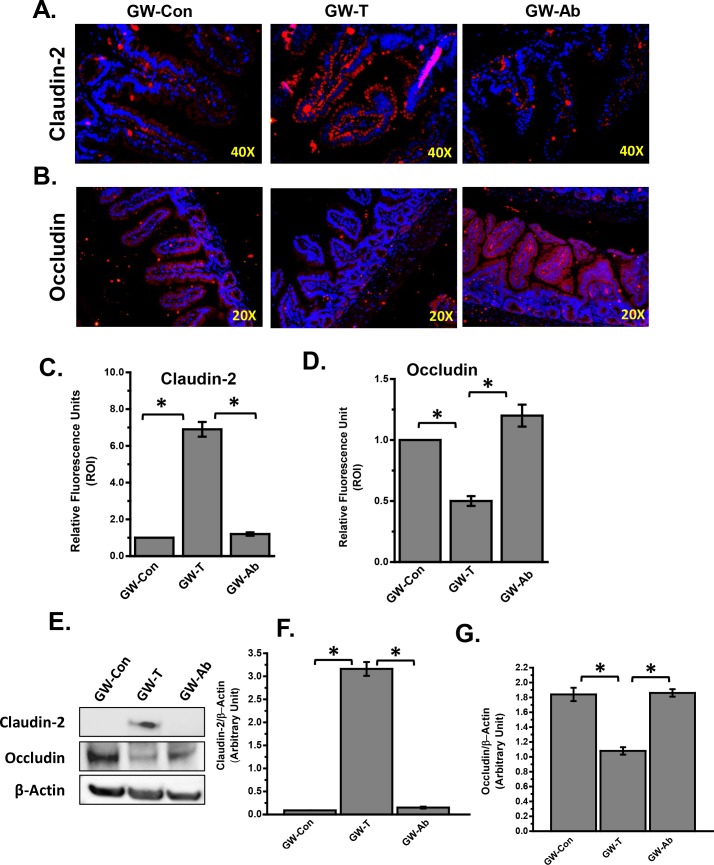
GW chemical-induced altered gut microbiome modulates gap junction protein levels in the small intestine. A. Tissue levels of Claudin-2 in control (GW-Con), treated (GW-T) and antibiotic treated (GW-Ab) samples as observed by immunofluorescent microscopy after labelling the protein with red fluorescent secondary antibody. Nuclear staining is shown by DAPI (blue) stain. B. Tissue levels of Occludin in control (GW-Con), treated (GW-T) and antibiotic treated (GW-Ab) samples as observed by immunofluorescent microscopy after labelling the protein with red fluorescent secondary antibody. Nuclear staining is shown by DAPI (blue) stain. C. Morphometry of fluorescence intensity as observed in the region of interest (ROI) Claudin-2 immunoreactivity and D. occludin immunoreactivity. E. Western blot analysis of Claudin-2 and Occludin in small intestine tissue homogenate. F-G. morphometric analysis of western blot. *(p<0.05). Data is represented as Mean+/- SE.

Several studies have shown that intestinal inflammation is accompanied by leaching of the gut resulting in flow of intestinal content including gut bacterial components in the portal circulation[48–52]. The “leaky gut” is often as a result of a significant decrease in tight junction proteins Claudin-1 and Occludin with an increase in Claudin-2[49]. Results showed that there was a significant increase in Claudin-2 and decrease in Occludin levels in the lower small intestinal segments in the GW-T group when compared to GW-Con groups (Fig 4A–4G). Administration of an antibiotic regimen of enrofloxacin and neomycin to achieve gut sterility and decontamination significantly decreased the Claudin-2 and increased Occludin protein levels as shown by immunofluorescence microscopy, western blot and subsequent morphometry of the images (Fig 4A–4G). The results suggested that altered gut microbiome in GW-T group with marked increases in Firmicutes (Phylum) and several genus such as *Allobaculum*, *Coprococcus*, *Dorea*, *Turibacter and Ruminococcus* that are known to be associated with inflammation, might play a significant role in expression of tight junction proteins Claudin-2 and Occludin.

### Altered gut microbial diversity in gulf war chemical exposed mice cause gut leaching and portal endotoxemia

**Fig 5:**
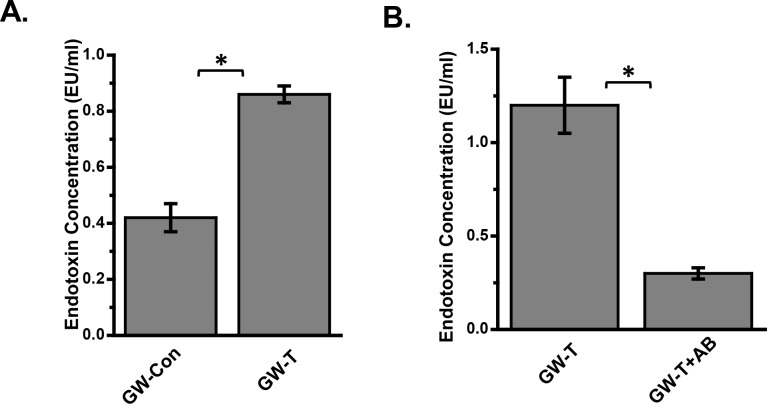
Portal endotoxemia following gulf war chemical exposure-induced altered gut microbiome. A. Endotoxin concentration as measured by **Limulus amebocyte lysate** (LAL) assay from serum of mice exposed to GW-chemicals. B. serum endotoxin levels in mice that were administered antibiotics (Neomycin and Enrofloxacin) to eliminate gut bacteria. *(p<0.05). Data is represented as Mean+/- SE.

The role of gut microbiota in various chronic inflammatory diseases and cancer has recently been established by extensive studies[53–55]. Several studies show an increase in endotoxin levels in experimental models upon changes in gut microbiome and enhanced intestinal permeability to endotoxins appears to be the primary cause of such systemic inflammation[56–61]. To study the role of gut microbiome changes-induced portal and systemic endotoxemia in gulf war chemical-exposed mice, blood endotoxin levels were analyzed by Limulus Amebocyte lysis (LAL) assay. Results showed that there was a significant increase (almost 2.0 fold) in systemic endotoxin levels in GW-T group as compared to GW-Con mice (Fig 5A)(P<0.05) while decontamination of the gastrointestinal tract with antibiotics in mice treated with antibiotics significantly decreased the endotoxin concentration in the blood (Fig 5B)(P<0.05). The results suggested that altered bacterial diversity and increase in certain bacterial genus might have resulted in the translocation of bacterial products into the systemic circulation. The translocation is possible via the intestinal tight junctions that have been rendered leaky following a significant decrease in Occludin and increase in Claudin-2 (Fig 4).

### Gut microbial changes, altered diversity in gulf war chemical exposure causes toll like receptor activation

**Fig 6:**
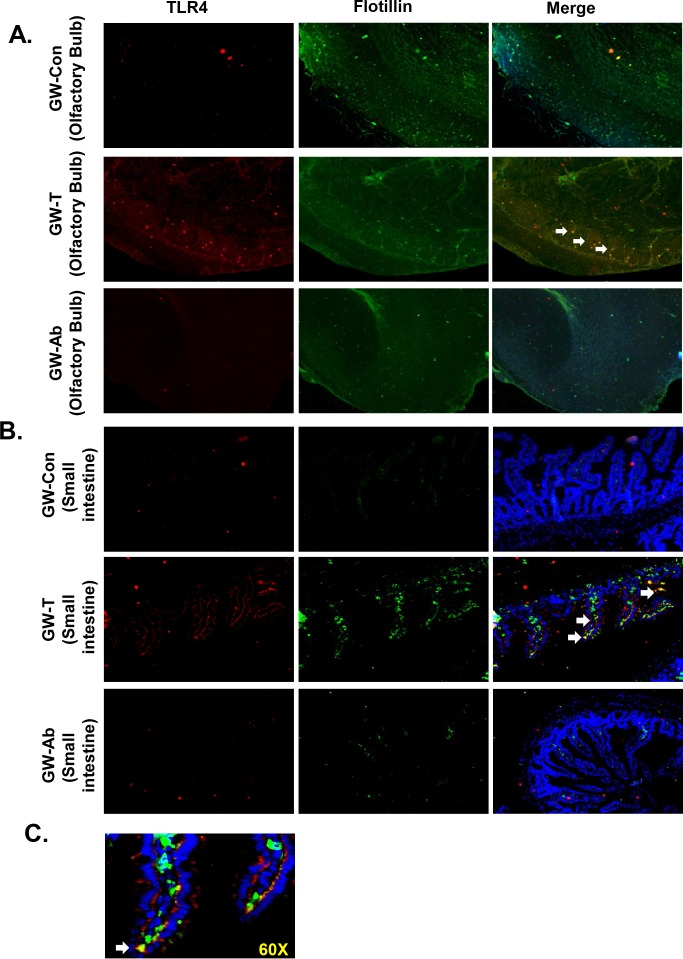
TLR4 activation following gulf war chemical exposure-induced altered gut microbiome. A. Immunofluorescence microscopy of olfactory bulb showing (TLR4 (red) trafficking to the lipid rafts (green), an essential process for TLR4 activation causing a co-localization of TLR4 in flotillin-rich rafts (yellow). B. Representative images of TLR4-flotillin co-localization in the small intestine shown by white arrow heads pointing to the yellow spots created by an overlay of red (TLR4) and green (Flotillin). C. Higher magnification (60x oil) representative image of the co-localization (TLR4 and Flotillin) in the small intestine from GW-treated samples.

**Fig 7:**
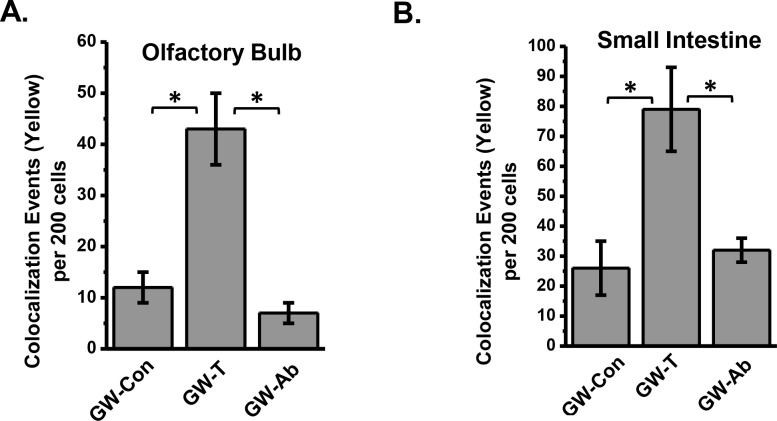
A. Graphical analysis of the number of co-localizations represented by yellow in individual groups of tissues (olfactory bulb) per 200 cells counted from randomly chosen microscopic fields. B. Graphical analysis of the number of co-localizations represented by yellow in individual groups of tissues (Small intestine) per 200 cells counted from randomly chosen microscopic fields *(p<0.05). Data is represented as Mean+/- SE.

To study the role of altered gut bacterial composition, resultant leaky gut and systemic endotoxemia in causing neuroinflammation and intestinal injury, studies were conducted to analyses the activation of TLR4 in frontal cortex, olfactory bulb and the ileum. TLR4 activation was analyzed by localizing the receptor in lipid rafts of the tissue plasma membrane. Results showed that the TLR4 co-localization in lipid rafts (trafficking) as shown by the increased number of colocalization events was significantly increased in olfactory bulb regions of the brain in the GW-T group when compared to GW-Con group (Figs 6A and 7A)(P<0.05). Decontamination of the intestinal tract by using an antibiotic regimen significantly decreased the TLR4 trafficking into the lipid rafts (Figs 6A and 7A). Similar significant increases were found in the intestinal segment of GW-T mice when compared with GW-Con group while treatment with antibiotics caused a significant decrease in the TLR4 trafficking when compared with the GW-T group (Figs 6B and 7B)(p<0.05). TLR4-Flotillin (lipid raft protein) colocalization was found in both the luminal side and the submucosal interface as shown by the higher magnification image (Fig 6C). The results suggested that bacterial content lipopolysaccharide might be the prime component in causing TLR4 activation in both brain and intestinal tissues. These observations further confirmed with LPS treated mice groups. The LPS exposed mice showed significant increase in TLR4 trafficking into the lipid rafts in both small intestine and olfactory bulb (Fig H in S1 File). This raises the possibility that altered gut microbiota, leaky gut and systemic endotoxemia might activate the TLR4 signaling pathway.

### TLR4 activation following gulf war chemical exposure-induced gut microbial changes cause neuronal and intestinal tyrosine nitration, a marker for both nitrative and oxidative stress

**Fig 8:**
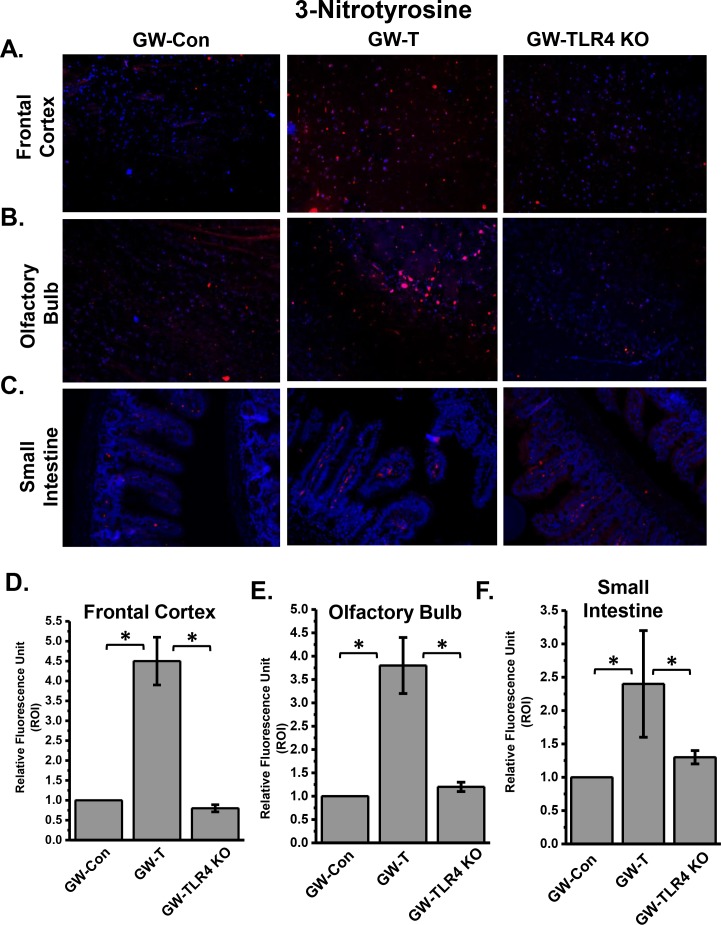
Oxidative stress as evidenced by tyrosine nitration of brain and intestinal tissues following GW chemical exposure and subsequent changes in the gut microbiome and TLR4 activation. A. Frontal cortex tissue slices showing immunoreactivity to 3-nitrotyrosine (red) followed by olfactory bulb (B) and small intestine(C). D, E and F shows the quantitative analysis of fluorescence intensities in the region of interest (ROI) in frontal cortex (D), Olfactory Bulb (E) and small intestine (F). *(p<0.05).

Oxidative and nitrative stress has been shown to be an integral cause of neuronal and intestinal inflammation[62–65]. Most often oxidative stress can result from an active TLR4 signaling pathway that causes NADPH oxidase activation and release of superoxide radicals[66, 67]. Nitric oxide is also released as a consequence of TLR4 activation and can react with superoxide in diffusion controlled rates to generate peroxynititre which in turn causes tyrosine nitration[66]. Results from immunofluorescence microscopy of brain and intestinal tissue slices probed with anti-3-nitrotyrosine antibodies showed that immunoreactivity of 3-nitrotyrosine in frontal cortex, olfactory bulb and small intestine significantly increased in GW-T group when compared to GW-Con group (Fig 8A–8F)(p<0.05) while use of TLR4 knockout mice or use of intestinal decontamination (data not shown) significantly decreased the formation of tyrosine nitration in these tissues (Fig 8A–8F)(p<0.05). The results suggested that the altered gut microbiome and the leaky gut caused increased oxidative stress through a TLR4 dependent pathway.

### TLR4 activation following gulf war chemical exposure-induced gut microbial changes cause neuronal and intestinal inflammation

**Fig 9:**
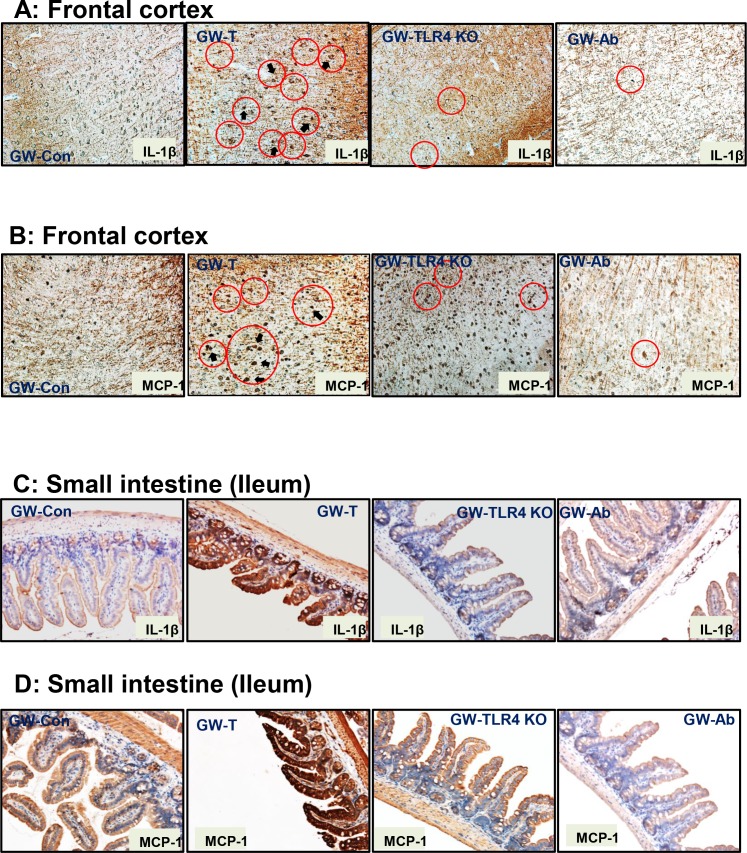
Gulf war chemical exposure-induced changes in gut microbiome modulates neuroinflammation and intestinal cytokine release. A. Frontal cortex tissue slices were probed for IL-1β immunoreactivity in control (GW-Con, treated (GW-T), TLR4 knockout mice treated with GW Chemical exposure (GW-TLR4KO) and antibiotic treated (GW-Ab) groups. Specific immunoreactivity to IL-1β as evident by dark brown spots are indicated by black arrows and areas of interest are outlined by red circles. B. Frontal cortex tissue slices were probed for MCP-1 immunoreactivity in control (GW-Con, treated (GW-T), TLR4 knockout mice treated with GW Chemical exposure (GW-TLR4KO) and antibiotic treated (GW-Ab) groups. Specific immunoreactivity to MCP-1 as evident by dark brown stain/spots are indicated by black arrows and areas of interest are outlined by red circles. C. Small intestine tissue slices were probed for IL-1β immunoreactivity in control (GW-Con, treated (GW-T), TLR4 knockout mice treated with GW Chemical exposure (GW-TLR4KO) and antibiotic treated (GW-Ab) groups. D. Small intestine tissue slices were probed for MCP-1 immunoreactivity in control (GW-Con, treated (GW-T), TLR4 knockout mice treated with GW Chemical exposure (GW-TLR4KO) and antibiotic treated (GW-Ab) groups.

**Fig 10:**
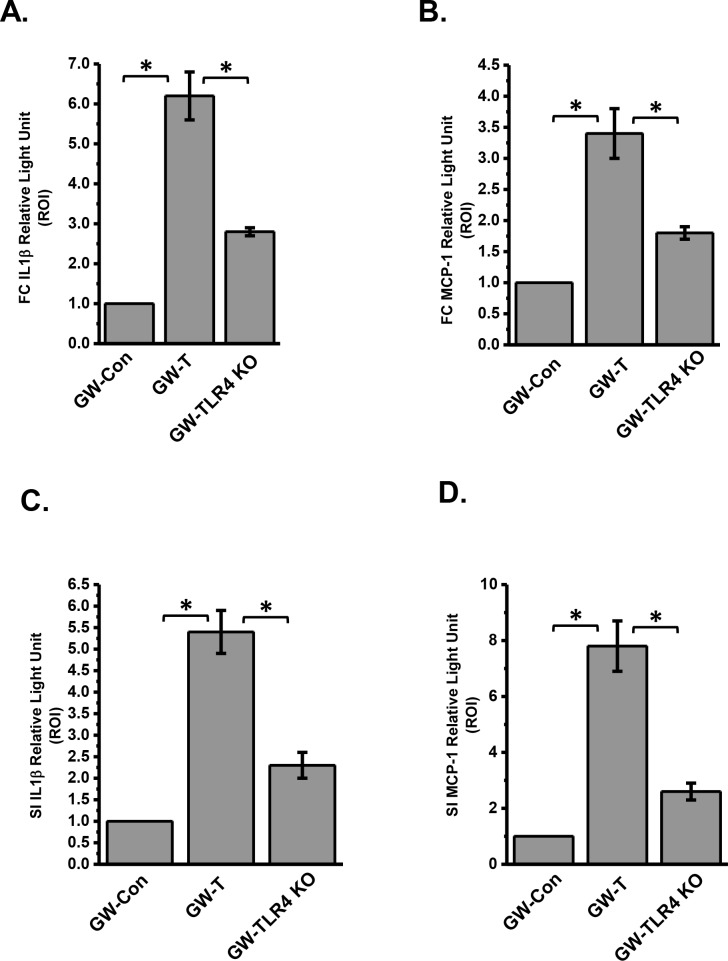
**A-D**. Morphometry of the immunoreactivities in tissue slices using the stained regions of interest. Data normalized against control and plotted in Microcal-Origin software *(p<0.05). Data is represented as Mean+/- SE.

Gulf war chemical exposure and rodent models of gulf war illness have shown increased levels of proinflammatory cytokines in the brain[10, 16, 68]. Interestingly inflammatory bowel disease also exhibits a strong inflammatory response primarily from high levels of IL1β and other chemokines[69]. To show that altered gut microbiome, a leaky gut and systemic endotoxemia result in local intestinal injury and an ectopic neural inflammation, tissue slices from the frontal cortex, and small intestine were probed for immunoreactivities against IL1β and monocyte chemoattractant protein-1 (MCP-1). Results showed that IL1β protein levels were significantly higher in the frontal cortex and small intestine segments of the GW-T group when compared to GW-Con group (Figs 9A, 9C, 10A and 10C)(p<0.05) while use of antibiotics for intestinal decontamination or TLR4 knockout mice showed a significant decrease in the immunoreactivities/protein levels of IL1β. Similar results were obtained for the protein levels of MCP-1 where the GW-T group had significantly higher levels of this chemokine as compared to GW-Con GW-Ab and TLR4 knockout mice groups. Other regions of the brain like cerebellum, hippocampus and hypothalamus were not examined. The results suggested that gulf war chemical exposure and the resultant changes in the gut microbial content had a key role to play in neuronal and intestinal inflammation primarily through a TLR4 dependent process.

## Discussion

**Fig 11:**
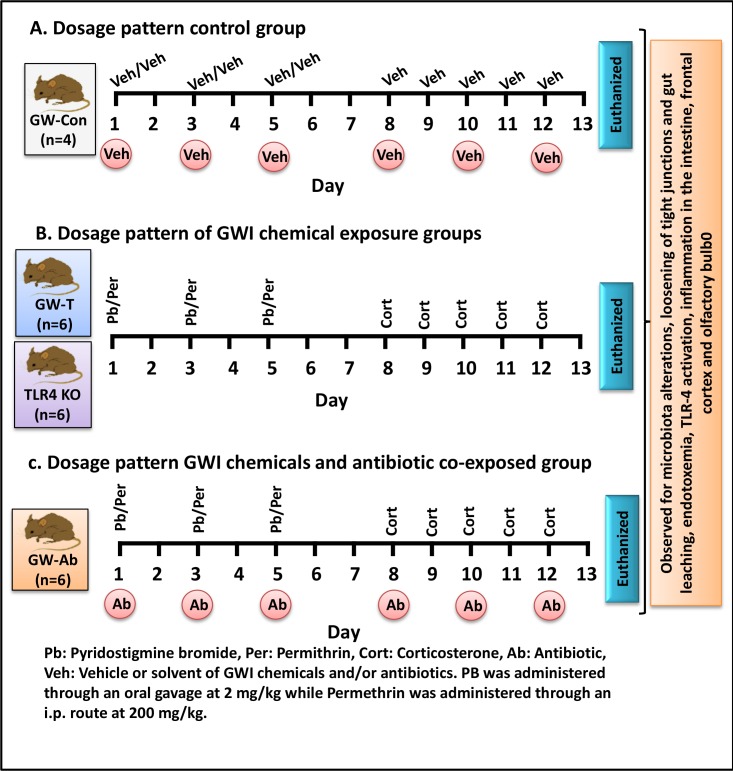
Schematic representation of dosage pattern: **A.** dosage pattern of control group (GW-Con) n = 4, C57BL/6J mice were exposed to vehicle/solvent (Veh) of the Gulf war chemicals and antibiotics. **B.** C57BL/6J (n = 6) and TLR4 KO (n = 6) mice were exposed to gulf war chemicals Pyridostigmine bromide (Pb), Permethrin (Per) and Corticosterone (Cort). **C.** C57BL/6J (n = 6) mice were co-exposed to gulf war chemicals (Pb, Per and Cort) and Antibiotics (Ab).

Our results show that regular consumption of pyridostigimine bromide (PB) and concomitant use of insecticide permethrin (as modelled in mice, Fig 11) in chronic stress alters the gut microbiome. The altered microbiome causes leaky gut and systemic endotoxemia leading to neuronal and intestinal inflammation. Recent studies in chronic inflammatory diseases like osteoarthritis, chronic kidney disease, inflammatory bowel syndrome report of altered microbial diversity[53–55, 70–72]. Obesity and nonalcoholic fatty liver disease that have significant low-lying inflammation have been associated with gut-microbial changes[53, 55].Systemic endotoxemia is known to activate Toll like receptor activation[73]. Often gram negative bacteria and its cell wall component lipopolysaccharide activate both TLR2 and TLR4 receptors in different organ systems. TLR4 activation is the most common occurrence with concomitant release of proinflammatory cytokines that are quintessential for inflammation[73, 74]. The present study is a novel report of a parallel mechanism (the other being the direct effects of this drug on the neuronal functions) of inflammation and tissue injury that can have far reaching effects on the health of gulf war veterans. The above results that provide a mechanistic input and a strong link to gut dysbiosis and inflammation and advance our understanding in other diseases like IBS, NAFLD and metabolic syndrome that altered microbiome can cause chronic inflammation to persist. The present study also throws light on possible microbiome-brain axis or vice versa for disease development in war veterans.

The present study showed a direct relationship of gulf war chemical exposure and altered gut microbiota diversity with changes in the Phylum and family levels. It is important to note that the phylum Firmicutes that has been found to be enriched in GW-T group is associated with obesity, metabolic syndrome, chronic fatigue and importantly inflammatory bowel disease, a common occurrence among gulf war veterans[57, 61, 75]. Our data also show that several bacterial family and genera were enriched in the GW-T group. They include Ruminoccoccaceae that has been associated with fibrosis, neuroinflammation and memory [76]. The present study also reports genus specific effects of bacteria in GW-T group that is associated in both small intestine and neuroinflammation[77]. *Allobaculum sp*. has been shown to be positively correlated with intestinal inflammation and a leaky gut (decreased tight junction proteins) [59]. *Coprococcus* and *Turicibacter* have been shown to be negatively correlated with disease severity and inflammation but these reports are contrary to what we found in our present study [69]. GW-T was highly correlated with neuroinflammation and gut leaching that had abundant *Coproccoccus* and *Turicibacter* (Fig 3). The apparent difference may be due to the exposure paradigm and disease type. The role pf PB in in causing neuroinflammation is unclear. Though GW-chemical exposure that included PB caused astrocytic activation and ICAM-1 increase a firm mechanistic argument in favor of the neuroinflammatory pathology is lacking[7]. [10, 68]. On the other hand PB did not show a significant increase in neuroinflammation[10]. Since stress alone can cause changes in the gut microbiome, the lack of stress-only group is a limitation in our studies. Though GWI is a combination of all the factors modelled, stress related altered microbial phenomenon can have tremendous implications. The present study identifies a specific role of gut microbiome in neuroinflammation and intestinal leakiness though it does not shed light on the blood brain barrier leakiness which is important to allow proinflammatory cytokines to target neuronal tissues. To address this specific question the present study hypothesized that it may not be the proinflammatory cytokines but high endotoxin concentration that bind to pattern recognition receptors in organ systems, in this case the brain and the small intestine, and cause a localized pro-inflammatory effect. Support for this hypothesis comes from the results that show an enrichment of gram negative bacteria in the GW-T group followed by a decrease in tight junction protein Occludin (Fig 4 and Fig 5). Interestingly, decreased levels of tight junction protein Occludin and increased Claudin-2 overwhelmingly show gut leakiness more often associated with IBS, NAFLD and alcoholic steatohepatitis[49, 52]. It is likely that in the present study, enriched gram negative bacteria and its components leaked into the systemic circulation and could access different organ systems including the brain. There are reports that brain uptake of circulating LPS is so low that most effects of peripherally administered LPS are likely mediated through LPS receptors located outside the blood brain barrier further confirming our argument that TLR4 activation is likely in this scenario [78]. A recent study also showed that the endothelial cells and perivascular macrophages at the blood brain barrier play a significant role in allowing immune signals enter the brain [79]. Further, neuroinflammation may be trigerred from other systemic inflammatory signals from the intestinal tract [80]. Evidence found in our study showed that toll like receptor 4, a pattern recognition receptor for the gram negative bacterial cell wall is activated in the small intestine, and the olfactory bulb (Fig 6A–6F). Though we did not study the immune signal flow at the blood brain barrier, an activation of the TLR4 via its trafficking to the lipid rafts is a strong indication that proinflammatory signals have reached the brain causing a significant increase in cytokines and chemokines. Our results further showed that IL1β and MCP-1 were significantly elevated in frontal cortex and small intestine along with tyrosine nitration (Figs 8 and 9 and 10). GWI is associated with chronic immune activation with higher levels of IL-1α, TNF-α, sTNFRII and IL-1β [10, 16]. Our results of increased protein levels of IL1β and MCP-1 in the frontal cortex and the small intestine show a progressive inflammatory pattern since the chemokine action of MCP-1 will further allow infiltration of leukocytes in the affected tissue[81]. This may be true to the intestine and is highly likely in the brain since activated microglia and resident cells expressing cytokines exist in that organ [82]. These cells can infiltrate brain tissue and can amplify the pro-inflammatory signal cascade initiated by the activation of TLR4 (Fig 6). However more studies with respect to neuroinflammation and the leaky gut in GWI will be required to definitively conclude the direct role of gut leaching in GWI.

Taken together, we present substantial evidence that might indicate an existence of an alternative immune network that arises from the ability of gulf war chemicals to alter the microbiome and enrich several gram-negative bacterial population. Further the altered microbiome-induced gut leakiness and endotoxemia might contribute to neuroinflammation and intestinal injury that are TLR4 dependent. The present study also is likely to support the notion that antibacterials like minocycline and probiotics might be a good therapeutic regimen in Gulf war illness and other associated disorders. The studies also might have tremendous translational impact since studies show that Ninety-nine percent of mouse genes are shared with humans at the host genetic level, and they share key similarities with the human gut microbiome at the phylum through family levels making them a powerful model system for evaluating host–microbiota interactions applicable to human biology [83]. However subtle differences in the gastrointestinal tract anatomy and corresponding bacterial species though small may influence the translational approach in future and requires cautious optimism.

## Supporting information

S1 File Supporting information. (DOCX)

S2 File Supporting tables. (DOCX)
