# Supplementary material for: Altered gut microbiome in a mouse model of Gulf War Illness causes neuroinflammation and intestinal injury via leaky gut and TLR4 activation
Source: PLoS One. 2017 Mar 22;12(3):e0172914. doi: 10.1371/journal.pone.0172914 (PMC5362211; doi:10.1371/journal.pone.0172914)
Supplement: S1 File — (DOCX) [file pone.0172914.s001.docx]

**S1 Text: Supplementary Information**

**Fig A**

**
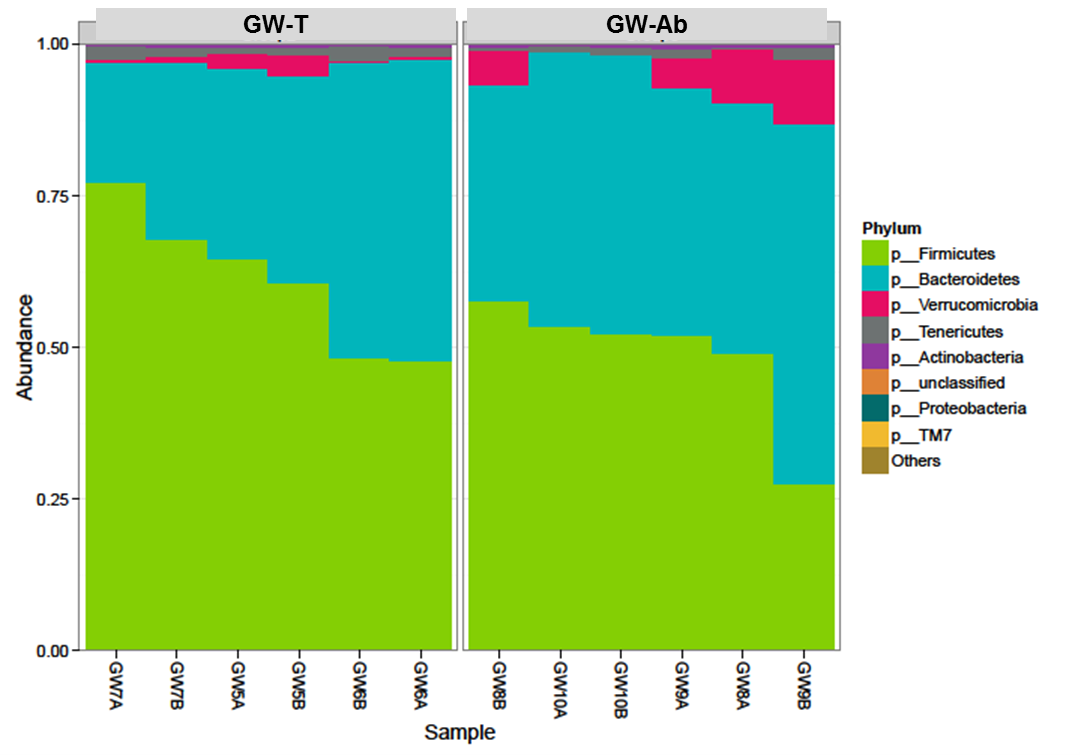
**

**Fig A. Gulf war chemical and antibiotic exposure alters gut microbiome at phylum level.** Proportional abundance of phyla: Graphical representation of the most abundant taxa of bacteria at the phylum level. Groups compared are gulf war chemical exposed group (GW-T, n=6) and gulf war chemical and antibiotic co-exposed group (GW-Ab, n=6). Groups include individual samples numbered at the time of V4 16S rRNA sequencing. Kruskal-Wallis rank sum test on top 8 most abundant phyla shows no significant relative Phylum-level abundance of phyla between the two groups GW-T and GW-Ab though there was a slight increase in Bacteridetes and a decrease in Firmecutes phyla in GW-Ab group..

**Table A: Kruskal-Wallis rank sum test on top 8 most abundant phyla. Percent relative abundance means are provided.**

**Phylum Chi-square KWPval GW-T Mean(sd) GW-Ab Mean(sd)**

**Firmicutes 1.641 0.20** **61.1(11.5) 48.7(10.7)**

**Bacteroidetes 1.641 0.20 35.5(11.7) 44.7(8.06)**

Verrucomicrobia 0.9231 0.34 1.38(1.31) 5.02(4.38)

Tenericutes 2.0769 0.15 1.65(0.514) 1.06(0.64)

Actinobacteria 2.5641 0.11 0.371(0.0723) 0.453(0.0981) unclassiﬁed 0 1.00 0.0269(0.00368) 0.0269(0.00329)

Proteobacteria 0.9231 0.34 0.014(0.0107) 0.015(0.00612)

TM7 2.1818 0.14 0.00111(0.00182) 0(0)

**Fig B**


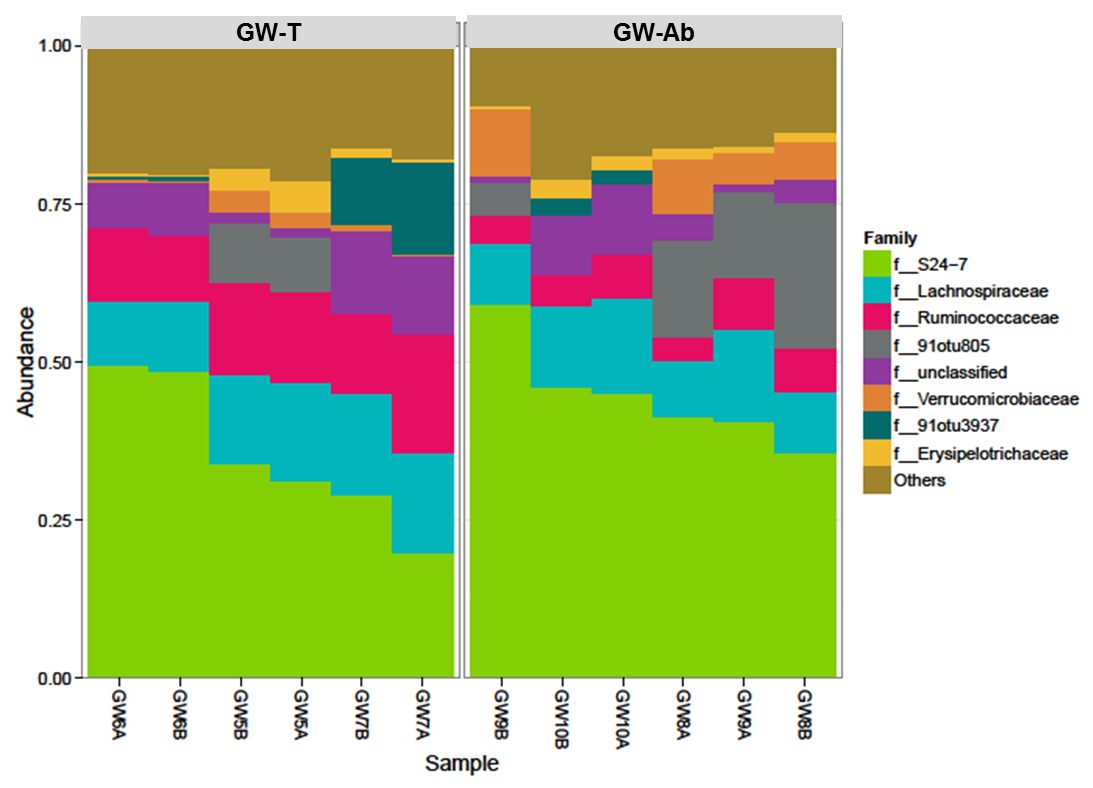


**Fig B. Gulf war chemical and antibiotic exposure alters gut microbiome at family level.** Proportional abundance of families: Graphical representation of most abundant taxa at the family level in gulf war chemical exposed group (GW-T, n=6) compared to gulf war chemical and antibiotic co-exposed group (GW-Ab, n=6). GW-T show a significant increase in relative abundance of Ruminococcaceae (KW p-value: <0.001) as compared with GW-Ab group.

**Table B: Kruskal-Wallis rank sum test on top 8 most abundant family. Percent relative abundance means are provided.**

| **Family** | **Chi-square** | **KWPval** | **GW-T Mean(sd)** | **GW-Ab Mean(sd)** |
| --- | --- | --- | --- | --- |
| S24-7 | 1.641 | 0.20 | 35.4(11.7) | 44.7(8.06) |
| Lachnospiraceae | 2.5641 | 0.11 | 13.8(2.63) | 11.8(2.73) |
| **Ruminococcaceae** | **8.3077** | **<0.001** | **13.7(2.87)** | **5.89(1.69)** |
| 91otu805 | 1.641 | 0.20 | 3.03(4.68) | 9.55(9.3) |
| unclassiﬁed | 0.9231 | 0.34 | 7.34(5.08) | 5.08(4.22) |
| Verrucomicrobiaceae | 0.9231 | 0.34 | 1.38(1.31) | 5.02(4.38) |
| 91otu3937 | 1.2564 | 0.26 | 4.34(6.38) | 0.824(1.27) |
| Erysipelotrichaceae | 0.1026 | 0.75 | 1.89(1.91) | 1.6(0.91) |


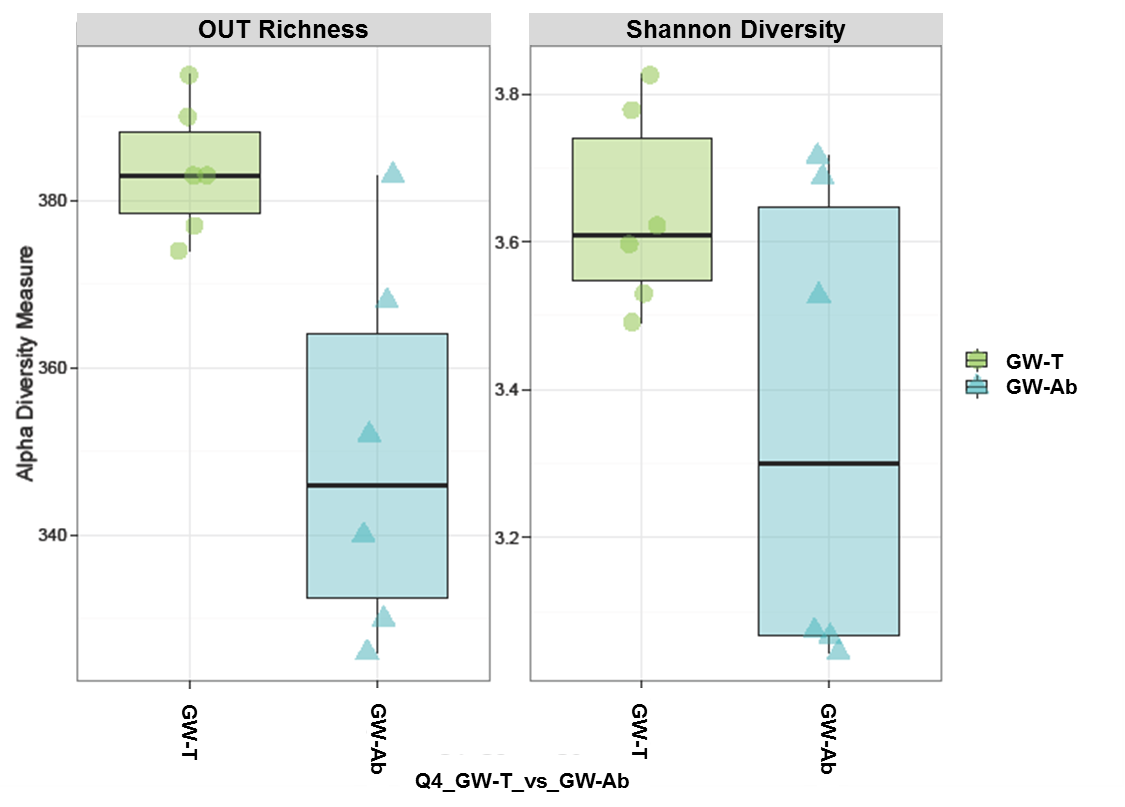


**Fig C. Alpha diversity estimates: Gulf war chemical exposure alters bacterial diversity in the intestinal lumen.** Left panel: OTU richness. Graphical representation of the number of OTUs present in each sample. Chao1 calculates the estimated sample richness (number of OTUs) based on sequencing depth and taking into account rare taxa that may be present in a samples of gulf war chemicals treated (GW-T) group has significant increase in OTU-richness compared to samples of gulf war chemicals and antibiotic co-exposed (GW-Ab) group (KW p-value: 0.02). Right panel: Graphical representation of Shannon diversity differences between GW-T and GW-Ab groups. Shannon diversity utilizes the richness of a sample along with the relative abundance of the present OTUs to calculate a diversity index. There is no observed significant increase in Shannon diversity of GW-T over GW-Ab.

**Table C: Kruskal-Wallis rank sum test on alpha diversity metrics**

| **Value** | | **Chi-square** | | **KWPval** | | **GW-T Mean(sd)** | | **GW-Ab Mean(sd)** |
| --- | --- | --- | --- | --- | --- | --- | --- | --- |
| OUT Richness | 5.8511 | | 0.02 | | 384(7.84) | | 350(22.3) | |
| Shannon Diversity | 2.0769 | | 0.15 | | 3.64(0.134) | | 3.35(0.325) | |


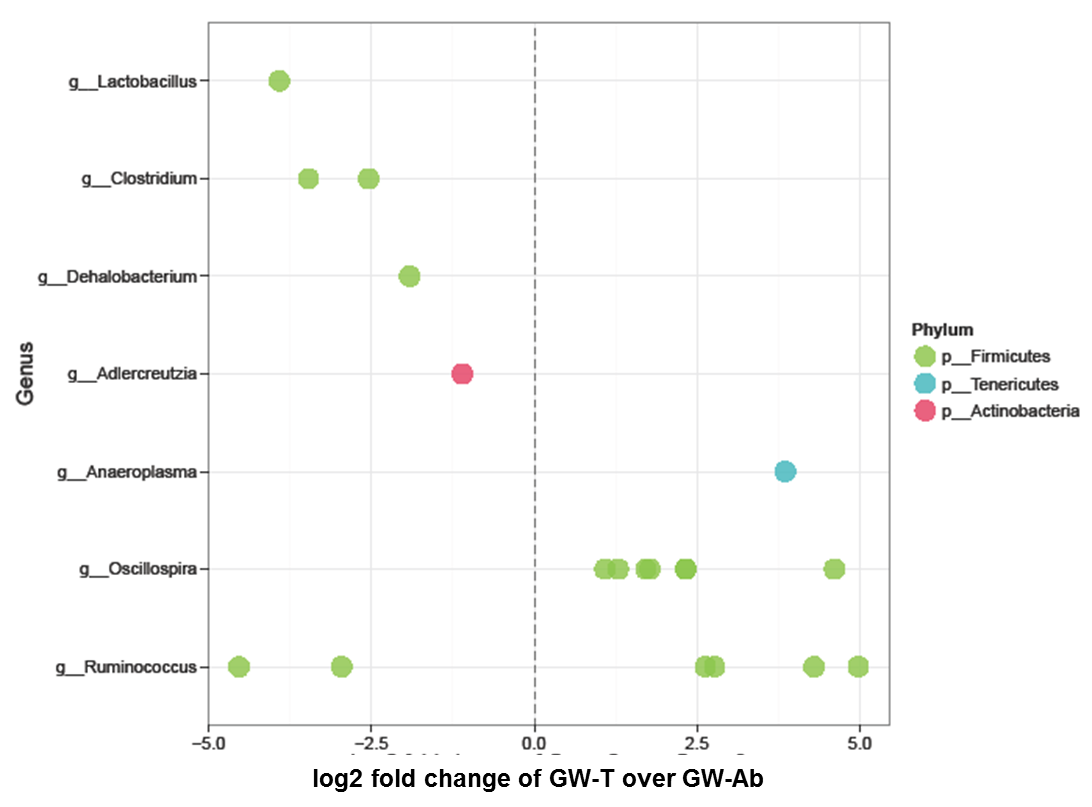


**Fig D. Differentially abundant features in gulf war chemical exposed group (GW-T) vs gulf war chemical and antibiotic co-exposed group (GW-Ab):** Each point represents an OTU belonging to each Genus. Features were considered significant if their FDR-corrected p-value was less than or equal to 0.05, and the absolute value of the Log-2 fold change was greater than or equal to 1. There were 75 significantly different features detected out of 600 tests. Only 19 features that were able to be classified at the genus level are shown in the plot.


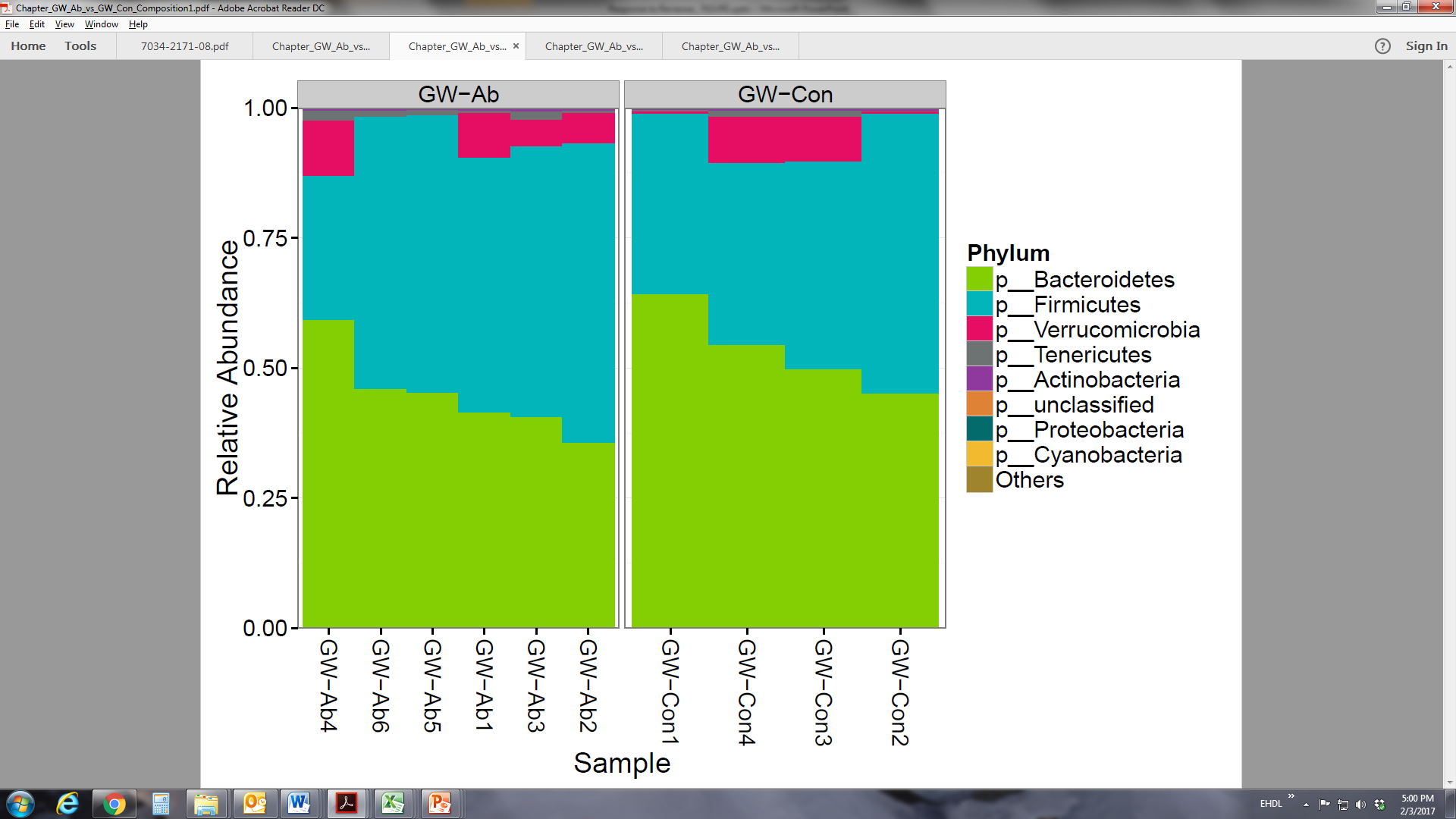


**Fig E. Antibiotic treated mice co-exposed with Gulf war chemical did not alters gut microbiome significantly as compared to control at phylum level.** Proportional abundance of phyla: Graphical representation of the most abundant taxa of bacteria at the phylum level. Groups compared are gulf war chemical control group (GW-Con, n=4) and gulf war chemical and antibiotic co-exposed group (GW-Ab, n=6). Groups include individual samples numbered at the time of V4 16S rRNA sequencing. Kruskal-Wallis rank sum test on top 8 most abundant phyla shows no significant relative Phylum-level abundance of phyla between the two groups GW-Con and GW-Ab as shown in table-S4.

**Table D: Kruskal-Wallis rank sum test on top 8 most abundant phyla. Percent relative abundance means are provided.**

| **Phylum** | **Chi-square** | **KW Pval** | **GW-Ab Mean (sd)** | **GW-Con Mean (sd)** |
| --- | --- | --- | --- | --- |
| p__Bacteroidetes | 2.2273 | 0.14 | 44.7 (8.06) | 53.4 (8.11) |
| p__Firmicutes | 0.7273 | 0.39 | 48.7 (10.7) | 40.8 (8.88) |
| p__Verrucomicrobia | 0.0455 | 0.83 | 5.02 (4.38) | 4.57 (4.81) |
| p__Tenericutes | 1.1364 | 0.29 | 1.06 (0.64) | 0.7 (0.556) |
| p__Actinobacteria | 0.1818 | 0.67 | 0.453 (0.0981) | 0.432 (0.0606) |
| p__unclassified | 0.7273 | 0.39 | 0.0269 (0.00329) | 0.0295 (0.00531) |
| p__Proteobacteria | 0.0455 | 0.83 | 0.015 (0.00612) | 0.0147 (0.00506) |
| p__Cyanobacteria | 1.5 | 0.22 | 0 (0) | 0.00026 (0.000519) |


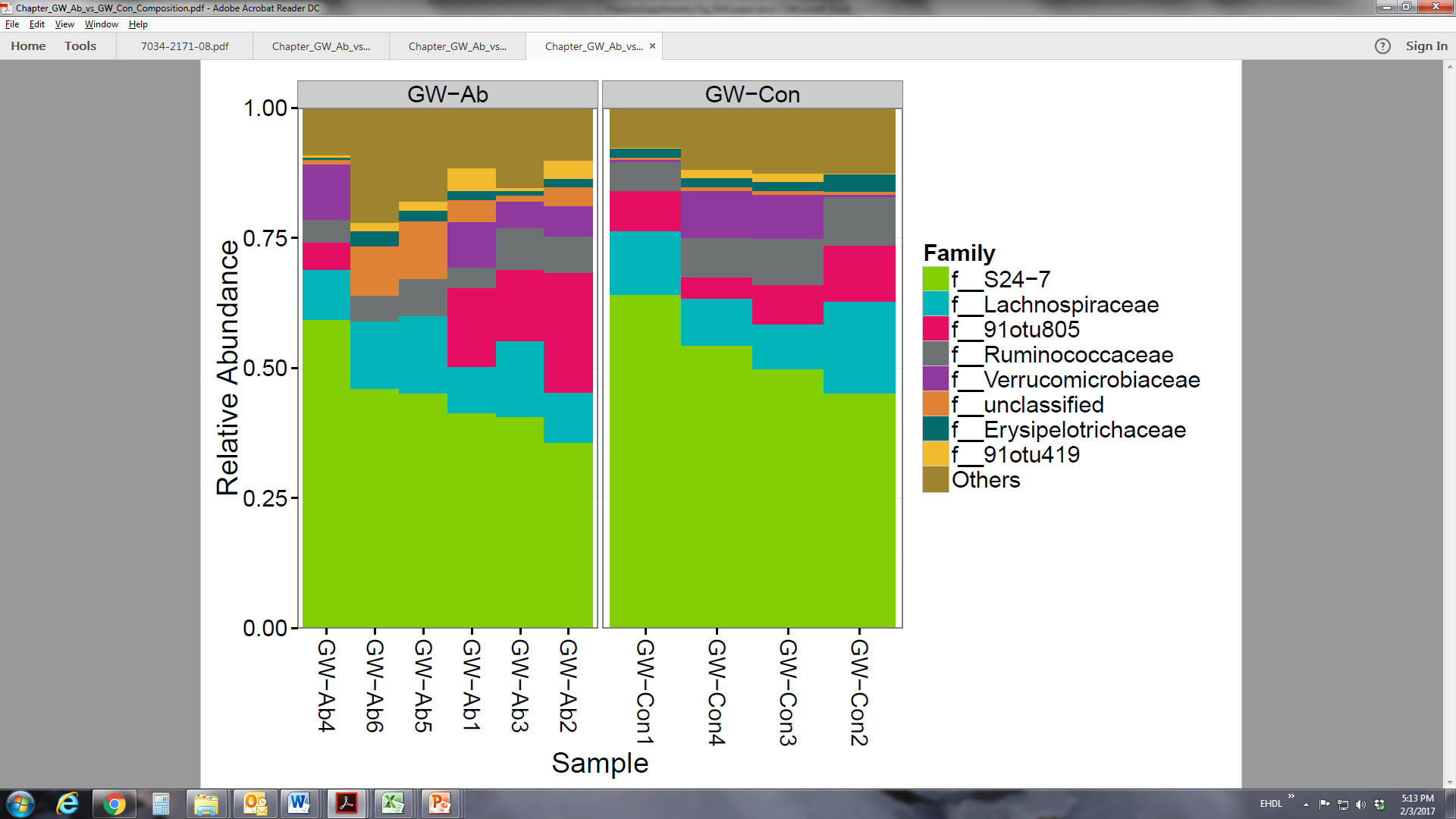


**Fig F. Antibiotic treated mice co-exposed with Gulf war chemical did not alters gut microbiome significantly as compared to control at family level.** Proportional abundance of families: Graphical representation of most abundant taxa at the family level in gulf war chemical control group (GW-Con, n=4) compared to gulf war chemical and antibiotic co-exposed group (GW-Ab, n=6). GW-Ab shows no significant increase in relative abundance of Ruminococcaceae as compared with GW-Con group. However, the one unclassified family has increased significantly in GW-Ab group as compared to GW-Con group (p=0.01) as in table-S5.

**Table E: Kruskal-Wallis rank sum test on top 8 most abundant families.** Percent relative abundance means are provided.

| **Family** | **Chi-square** | **KW Pval** | **GW-Ab Mean (sd)** | **GW-Con Mean (sd)** |
| --- | --- | --- | --- | --- |
| f__S24-7 | 2.2273 | 0.14 | 44.7 (8.06) | 53.4 (8.11) |
| f__Lachnospiraceae | 0.1818 | 0.67 | 11.8 (2.73) | 11.8 (4.2) |
| f__91otu805 | 0.0455 | 0.83 | 9.55 (9.3) | 7.57 (2.74) |
| f__Ruminococcaceae | 2.9091 | 0.09 | 5.89 (1.69) | 7.81 (1.65) |
| f__Verrucomicrobiaceae | 0.0455 | 0.83 | 5.02 (4.38) | 4.57 (4.81) |
| **f__unclassified** | **6.5455** | **0.01** | **5.08 (4.22)** | **0.619 (0.156)** |
| f__Erysipelotrichaceae | 1.6364 | 0.2 | 1.6 (0.91) | 2.18 (0.796) |
| f__91otu419 | 2.9091 | 0.09 | 2.03 (1.66) | 0.866 (0.795) |


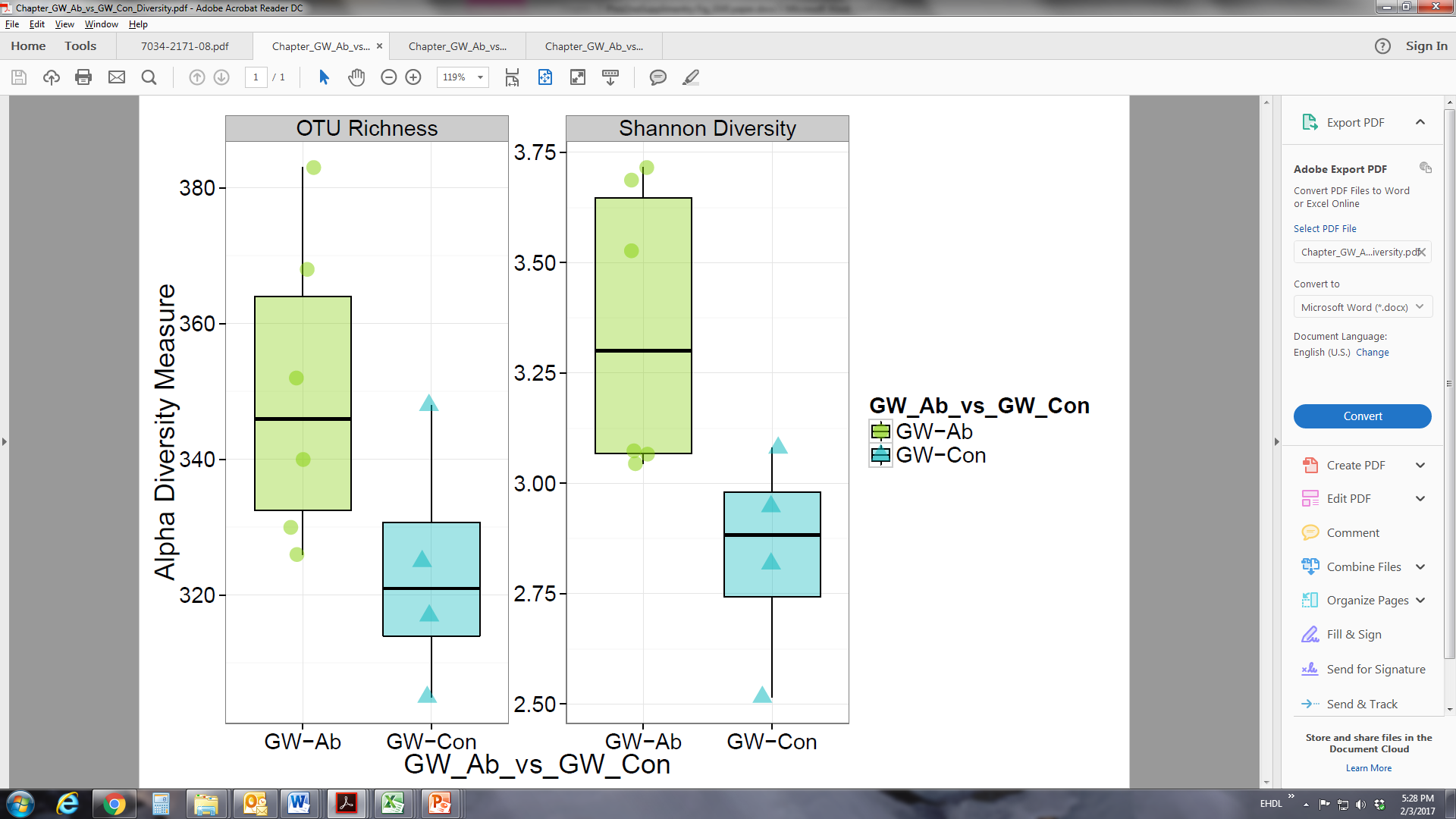


**Fig G. Alpha diversity estimates: Left panel**: OTU richness. Graphical representation of the number of OTUs present in each sample. Chao1 calculates the estimated sample richness (number of OTUs) based on sequencing depth and taking into account rare taxa that may be present in samples of gulf war chemicals control (GW-Con) groups and gulf war chemicals and antibiotic co-exposed (GW-Ab) groups. As observed, the increased OTU-richness in GW-Ab group is not significant when compared to GW-Con groups (Table S6). **Right panel:** Graphical representation of Shannon diversity differences between GW-Con and GW-Ab groups. Shannon diversity utilizes the richness of a sample along with the relative abundance of the present OTUs to calculate a diversity index. There is no observed significant increase in Shannon diversity of GW-Con over GW-Ab (Table S6).

**Table F: Kruskal-Wallis rank sum test on alpha diversity metrics**

| **Value** | **Chi-square** | **KW Pval** | **GW-Ab Mean (sd)** | **GW-Con Mean (sd)** |
| --- | --- | --- | --- | --- |
| OTU Richness | 3.6818 | 0.06 | 350 (22.3) | 324 (18.1) |
| Shannon Diversity | 3.6818 | 0.06 | 3.35 (0.325) | 2.84 (0.242) |


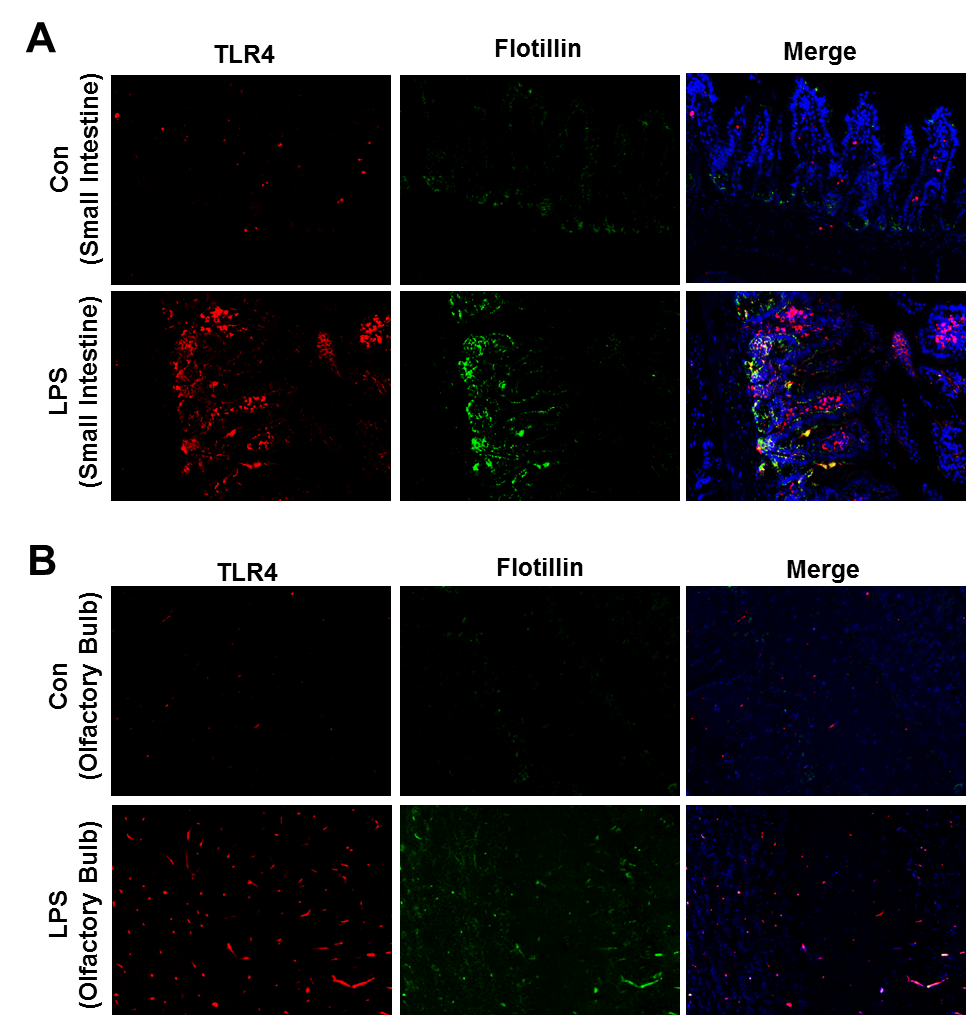


**Fig H. TLR4 activation induced by Lipopolysaccharide (LPS) exposure.** A. Immunofluorescence microscopy of small intestine showing TLR4 (red) trafficking to the lipid rafts an indicated by flotillin (green), an essential process for TLR4 activation causing a co-localization of TLR4 in flotillin-rich rafts (yellow) in control group (n=3) and LPS (12mg/kg) treated group. B. Representative images of TLR4-flotillin co-localization in the olfactory bulb of control group and LPS treated group.

Raw data for Western Blot quantification:

| **Occludin** |  | Intensity1 | Intensity2 | Intensity3 | Average |
| --- | --- | --- | --- | --- | --- |
|  | GW-Con | 23930 | 27510 | 26335 | 25925 |
|  | GW-T | 12012 | 13458 | 10380 | 11950 |
|  | GW-Ab | 15898 | 15041 | 17010 | 15983 |
|  |  |  |  |  |  |
| **Claudin-2** |  | Intensity1 | Intensity2 | Intensity3 | Average |
|  | GW-Con | 88 | 92 | 120 | 100 |
|  | GW-T | 36158 | 34985 | 33764 | 34969 |
|  | GW-Ab | 1321 | 1229 | 1412 | 1320.667 |
|  |  |  |  |  |  |
| **β-Actin** |  | Intensity1 | Intensity2 | Intensity3 | Average |
|  | GW-Con | 13401 | 13991 | 14854 | 14082 |
|  | GW-T | 11086 | 10295 | 11790 | 11057 |
|  | GW-Ab | 8320 | 8267 | 9162 | 8583 |

**Data for Fig 4F and 4G.**

Raw data for microbiome analysis : Accession number: **PRJEB19474**

The quantitation data and accession number of microbiome data deposited in NCBI/EBI is **PRJEB19474**
