# Supplementary material for: Altered gut microbiome in a mouse model of Gulf War Illness causes neuroinflammation and intestinal injury via leaky gut and TLR4 activation
Source: PLoS One. 2017 Mar 22;12(3):e0172914. doi: 10.1371/journal.pone.0172914 (PMC5362211; doi:10.1371/journal.pone.0172914)
Supplement: S2 File — (DOCX) [file pone.0172914.s002.docx]

**Table A: Kruskal-Wallis rank sum test on top 8 most abundant phyla. Percent relative abundance means are provided.**

**Phylum Chi-square KWPval GW-T Mean(sd) GW-Ab Mean(sd)**

**Firmicutes 1.641 0.20** **61.1(11.5) 48.7(10.7)**

**Bacteroidetes 1.641 0.20 35.5(11.7) 44.7(8.06)**

Verrucomicrobia 0.9231 0.34 1.38(1.31) 5.02(4.38)

Tenericutes 2.0769 0.15 1.65(0.514) 1.06(0.64)

Actinobacteria 2.5641 0.11 0.371(0.0723) 0.453(0.0981) unclassiﬁed 0 1.00 0.0269(0.00368) 0.0269(0.00329)

Proteobacteria 0.9231 0.34 0.014(0.0107) 0.015(0.00612)

TM7 2.1818 0.14 0.00111(0.00182) 0(0)

**Table B: Kruskal-Wallis rank sum test on top 8 most abundant family. Percent relative abundance means are provided.**

| **Family** | **Chi-square** | **KWPval** | **GW-T Mean(sd)** | **GW-Ab Mean(sd)** |
| --- | --- | --- | --- | --- |
| S24-7 | 1.641 | 0.20 | 35.4(11.7) | 44.7(8.06) |
| Lachnospiraceae | 2.5641 | 0.11 | 13.8(2.63) | 11.8(2.73) |
| **Ruminococcaceae** | **8.3077** | **<0.001** | **13.7(2.87)** | **5.89(1.69)** |
| 91otu805 | 1.641 | 0.20 | 3.03(4.68) | 9.55(9.3) |
| unclassiﬁed | 0.9231 | 0.34 | 7.34(5.08) | 5.08(4.22) |
| Verrucomicrobiaceae | 0.9231 | 0.34 | 1.38(1.31) | 5.02(4.38) |
| 91otu3937 | 1.2564 | 0.26 | 4.34(6.38) | 0.824(1.27) |
| Erysipelotrichaceae | 0.1026 | 0.75 | 1.89(1.91) | 1.6(0.91) |

**Table C: Kruskal-Wallis rank sum test on alpha diversity metrics**

| **Value** | | **Chi-square** | | **KWPval** | | **GW-T Mean(sd)** | | **GW-Ab Mean(sd)** |
| --- | --- | --- | --- | --- | --- | --- | --- | --- |
| OUT Richness | 5.8511 | | 0.02 | | 384(7.84) | | 350(22.3) | |
| Shannon Diversity | 2.0769 | | 0.15 | | 3.64(0.134) | | 3.35(0.325) | |

**Table D: Kruskal-Wallis rank sum test on top 8 most abundant phyla. Percent relative abundance means are provided.**

| **Phylum** | **Chi-square** | **KW Pval** | **GW-Ab Mean (sd)** | **GW-Con Mean (sd)** |
| --- | --- | --- | --- | --- |
| p__Bacteroidetes | 2.2273 | 0.14 | 44.7 (8.06) | 53.4 (8.11) |
| p__Firmicutes | 0.7273 | 0.39 | 48.7 (10.7) | 40.8 (8.88) |
| p__Verrucomicrobia | 0.0455 | 0.83 | 5.02 (4.38) | 4.57 (4.81) |
| p__Tenericutes | 1.1364 | 0.29 | 1.06 (0.64) | 0.7 (0.556) |
| p__Actinobacteria | 0.1818 | 0.67 | 0.453 (0.0981) | 0.432 (0.0606) |
| p__unclassified | 0.7273 | 0.39 | 0.0269 (0.00329) | 0.0295 (0.00531) |
| p__Proteobacteria | 0.0455 | 0.83 | 0.015 (0.00612) | 0.0147 (0.00506) |
| p__Cyanobacteria | 1.5 | 0.22 | 0 (0) | 0.00026 (0.000519) |

**Table E: Kruskal-Wallis rank sum test on top 8 most abundant families.** Percent relative abundance means are provided.

| **Family** | **Chi-square** | **KW Pval** | **GW-Ab Mean (sd)** | **GW-Con Mean (sd)** |
| --- | --- | --- | --- | --- |
| f__S24-7 | 2.2273 | 0.14 | 44.7 (8.06) | 53.4 (8.11) |
| f__Lachnospiraceae | 0.1818 | 0.67 | 11.8 (2.73) | 11.8 (4.2) |
| f__91otu805 | 0.0455 | 0.83 | 9.55 (9.3) | 7.57 (2.74) |
| f__Ruminococcaceae | 2.9091 | 0.09 | 5.89 (1.69) | 7.81 (1.65) |
| f__Verrucomicrobiaceae | 0.0455 | 0.83 | 5.02 (4.38) | 4.57 (4.81) |
| **f__unclassified** | **6.5455** | **0.01** | **5.08 (4.22)** | **0.619 (0.156)** |
| f__Erysipelotrichaceae | 1.6364 | 0.2 | 1.6 (0.91) | 2.18 (0.796) |
| f__91otu419 | 2.9091 | 0.09 | 2.03 (1.66) | 0.866 (0.795) |

**Table F: Kruskal-Wallis rank sum test on alpha diversity metrics**

| **Value** | **Chi-square** | **KW Pval** | **GW-Ab Mean (sd)** | **GW-Con Mean (sd)** |
| --- | --- | --- | --- | --- |
| OTU Richness | 3.6818 | 0.06 | 350 (22.3) | 324 (18.1) |
| Shannon Diversity | 3.6818 | 0.06 | 3.35 (0.325) | 2.84 (0.242) |
